# Identification of Lymph Node Metastasis–Related Key Genes and Prognostic Risk Model in Bladder Cancer by Co-Expression Analysis

**DOI:** 10.3389/fmolb.2021.633299

**Published:** 2021-07-22

**Authors:** Cheng Luo, Bin Huang, Yukun Wu, Yadong Xu, Wei Ou, Junxing Chen, Lingwu Chen

**Affiliations:** Department of Urology, The First Affiliated Hospital, Sun Yat-Sen University, Guangzhou, China

**Keywords:** bladder cancer, weighted gene co-expression network analysis, hub genes, lymph node metastasis, prognostic signature

## Abstract

**Background:** Lymph node metastasis (LNM) is an important pathological characteristic of bladder cancer (BCa). However, the molecular mechanism underlying LNM was not thoroughly elaborated. Identification for LNM-related biomarkers may contribute to making suitable therapies. So, the current study was aimed to identify key genes and construct a prognostic signature.

**Methods:** Based on the Cancer Genome Atlas (TCGA) database, gene expression and clinical information were obtained. Then, the weighted gene co-expression network analysis (WGCNA) was performed to identify the key modules and hub genes. A function analysis and a gene set enrichment analysis were applied to explore biological functions and pathways of interested genes. Furthermore, a prognostic model based on LNM-related genes was constructed by using the least absolute shrinkage and selection operator (LASSO) Cox regression analysis.

**Results:** Finally, nine co-expression modules were constructed, and two modules (turquoise and green) were significantly associated with LNM. Three hub genes were identified as DACT3, TNS1, and MSRB3, which were annotated in actin binding, actin cytoskeleton, adaptive immune response, and cell adhesion molecular binding by the GSEA method. Further analysis demonstrated that three hub genes were associated with the overall survival of BCa patients. In addition, we built a prognostic signature based on the genes from LNM-related modules and evaluated the prognostic value of this signature.

**Conclusion:** In general, this study revealed the key genes related to LNM and prognostic signature, which might provide new insights into therapeutic target of BCa.

## Introduction

Bladder cancer (BCa) is the second most common malignancy of the genitourinary cancers (Siegel et al., 2019). Approximately 25% of BCa in patients are pathologically defined as muscle-invasive tumors. Pelvic lymph nodes are commonly the first site for metastasis of BCa (Witjes et al., 2020). Radical cystectomy with lymph node dissection is the standard treatment for these patients, of whom 18.0–30.4% harbored nodal metastasis (Youssef and Raj, 2011; Hautmann et al., 2012). Lymph node dissection provides accurate node staging and predictive information for oncologic outcomes except for the tumor stage and grade. In addition, several parameters, based on the lymph node status including positive node count, lymph node density, and log odds, were proposed as prognostic factors for BCa patients (Fransen van de Putte et al., 2015; Masson-Lecomte et al., 2013; Lee et al., 2012; Jin et al., 2019).

Significant differences regarding to the prognosis of patients with positive and negative lymph nodes have been observed in previous studies (Al-Daghmin et al., 2014; Cha et al., 2018). A recent study revealed that NONO, acting as a splicing factor, could regulate the splicing of SETMER to suppress the lymphatic metastasis of BCa (Xie et al., 2021). However, the explicit molecular mechanisms behind the clinical phenomenon remain to be explored. In recent decade, due to the wide application of the high-throughput sequencing technology, gene signatures on the basis of genetic network were proposed to be accurate models to evaluate the malignancy and predict prognosis in many tumors (Zhang et al., 2018; Sun et al., 2020). A weighted gene co-expression network analysis (WGCNA) is a biological algorithm that investigates the inherent characteristics of gene sets and figures out the relationships between phenotypes and gene modules (Clarke et al., 2013). Functional gene modules are applied to screen out hub genes as potential biomarkers and therapeutic targets (Huang et al., 2017).

In the present study, we aimed to identify hub genes associated with lymph node metastasis (LNM) in BCa and explore novel gene markers to build a prognostic model. Using co-expression network analysis based on the WGCNA algorithm, several gene modules and hub genes were screened out, which might provide novel insight in the therapeutic exploration for BCa patients with LNM.

## Materials and Methods

### Data Acquisition and DEGs Screening

We downloaded the gene expression data and clinical information of 409 BCa and 19 normal samples from the GDC data portal of the Cancer Genome Atlas (TCGA) database (https://cancergenome.nih.gov/). In addition, gene expression profiles and survival data of 165 primary bladder cancer of the GSE13507 were downloaded from the GEO database (http://www.ncbi.nlm.nih.gov/geo/). Before the WGCNA analysis, the expression level of each gene was log2 transformed, and the calculation of the average value was adopted for multiple probes corresponding to the same gene. Then, “Limma” package in the R language environment was applied for the screening of differentially expressed genes (DEGs) according to the criteria of false discovery rate (FDR) < 0.05 and absolute of log2 fold change > 1. Then, samples with gene expression and integrated information of lymph node status were included for further co-expression analysis.

### Weighted Gene Co-Expression Network Analysis

Screened DEGs were prepared for further scale-free network construction, which was built on the WGCNA algorithm in R project (Langfelder and Horvath, 2008). First, the appropriate soft threshold power *β* was determined by using the pickSoftThreshold function. Then, Pearson’s correlations of DEGs were calculated to evaluate the similarity of gene expression patterns to build an adjacency matrix. After the gene co-expression network was completed, we utilized the dynamic tree cut algorithm to detect the modules. The parameters included were 30 for a minimum module size and 0.25 for merged cut height. Finally, modules conforming to these criteria were determined, and the dendrogram was generated accordingly.

### Identification of Clinically Significant Modules and Functional Enrichment Analysis

Module eigengene, which reflected the expression level of genes within a particular module, was the major principle of this module. The association between module eigengene and clinical traits including LNM was evaluated by Pearson’s correlation tests and presented as gene significance. We selected those modules with significant correlation (*p*-value < 0.01) with LNM for subsequent functional enrichment analysis. Then, genes in modules of interest were put into the Database for Annotation, Visualization, and Integrated Discovery (DAVID, http://david.ncifcrf.gov/) website for Gene Ontology (GO) enrichment and the Kyoto Encyclopedia of Genes and Genomes (KEGG) pathway analysis. The results were visualized by “stringr” and “ggplot2” packages.

### Identification of Hub Genes and Gene Set Enrichment Analysis

The module with the highest Pearson’s correlation coefficient was selected for screening of hub genes. LNM-related genes were imported into Cytoscape software, and then the network was constructed. Next, top ten nodes were identified through the calculation ranked by degree. Furthermore, three core genes in this critical network were identified and selected for subsequent GSEA, which was performed by using “clusterProfiler” R package. For each core gene, five enrichment results were presented for GO and KEGG analyses.

### Clinical Correlation and Prognostic Signature Construction

The expression levels of ten core genes in negative and positive lymph node groups were compared. By applying the median value as the cutoff point, the prognostic impact of core genes on BCR-free survival was evaluated. To further construct a prognostic signature based on LNM-related module, the least absolute shrinkage and selection operator (LASSO) regression was applied to filter prognostic genes. Then, a prognostic signature was built on the basis of regression coefficients from the multivariate COX regression analysis. The signature score was calculated as follows: (exprgene1 × Coefgene1) + (exprgene2 × Coefgene2) + …… + (exprgenen × Coefgenen). According to the median value of the signature score as the cutoff point, patients were classified as high- and low-risk groups. The Kaplan–Meier method, time-dependent receiver operating characteristic (ROC) curve, and the multivariate COX regression analysis were applied to evaluate the prognostic efficiency of the signature. The prognostic model was validated internally with randomly selected 40% of TCGA dataset and externally with GSE13507 from GEO dataset.

### Statistical Analysis

The gene expression levels in negative and positive lymph node groups were compared by the nonparametric Mann–Whitney U test. The Kaplan–Meier method and the log-rank test were applied to evaluate the prognostic impact of core genes and signature score on the overall survival. The WGCNA analysis was performed by the Pearson correlation analysis. All of these procedures were conducted by R software (version 3.6.2). *p*-value < 0.05 was defined statistically significant.

## Results

### Construction of Weighted Co-expression Network and Identification of Key Modules

**FIGURE 1:**
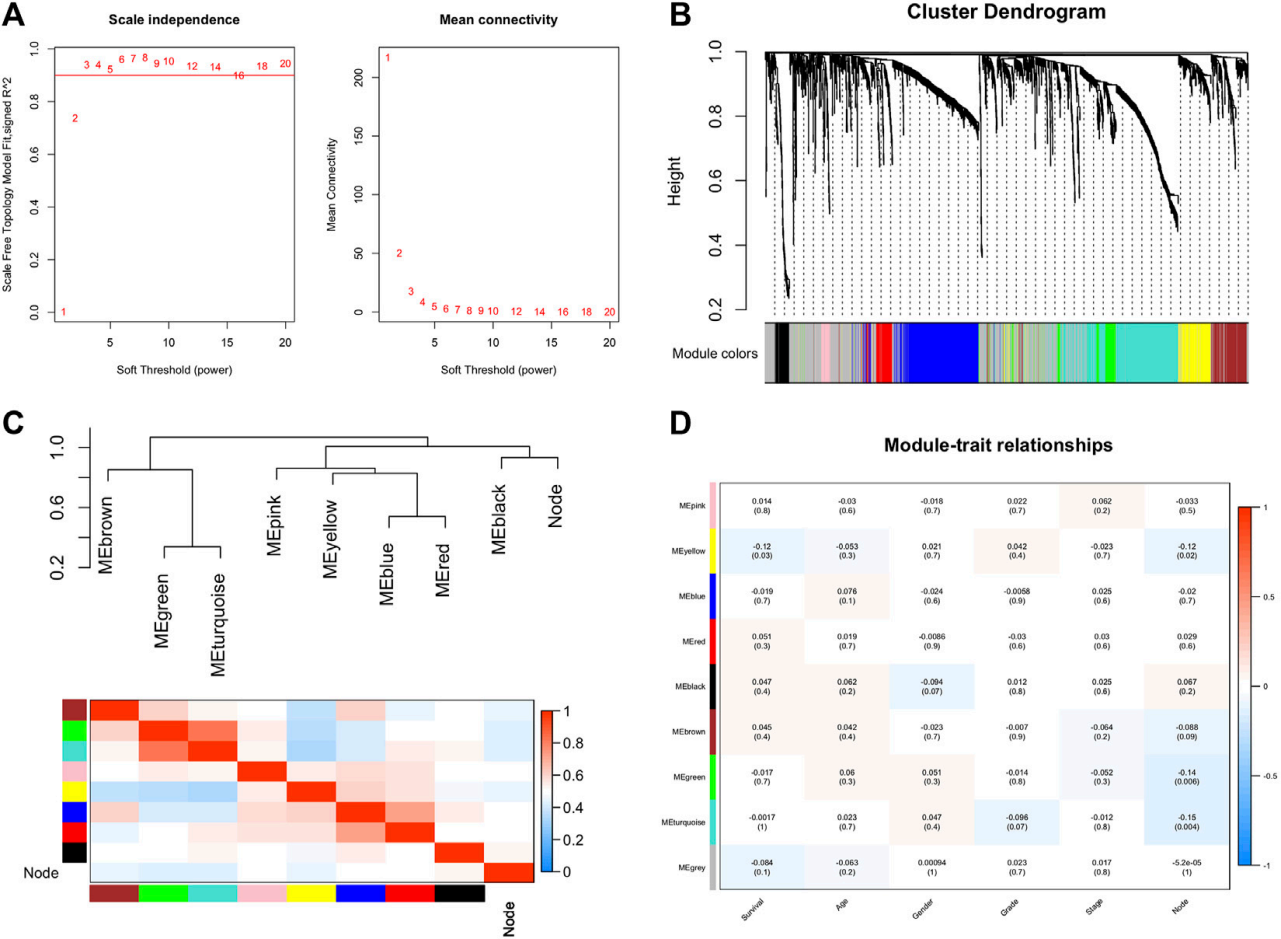
Construction of co-expression network through WGCNA. **(A)** Network topology for different soft-thresholding powers. **(B)** A cluster diagram of gene cluster of bladder cancer. **(C)** Heatmap of the adjacencies in the hub gene network. **(D)** Heatmap of the correlation between module eigengenes and the clinical features. WGCNA: weighted gene co-expression network analysis.

**FIGURE 2:**
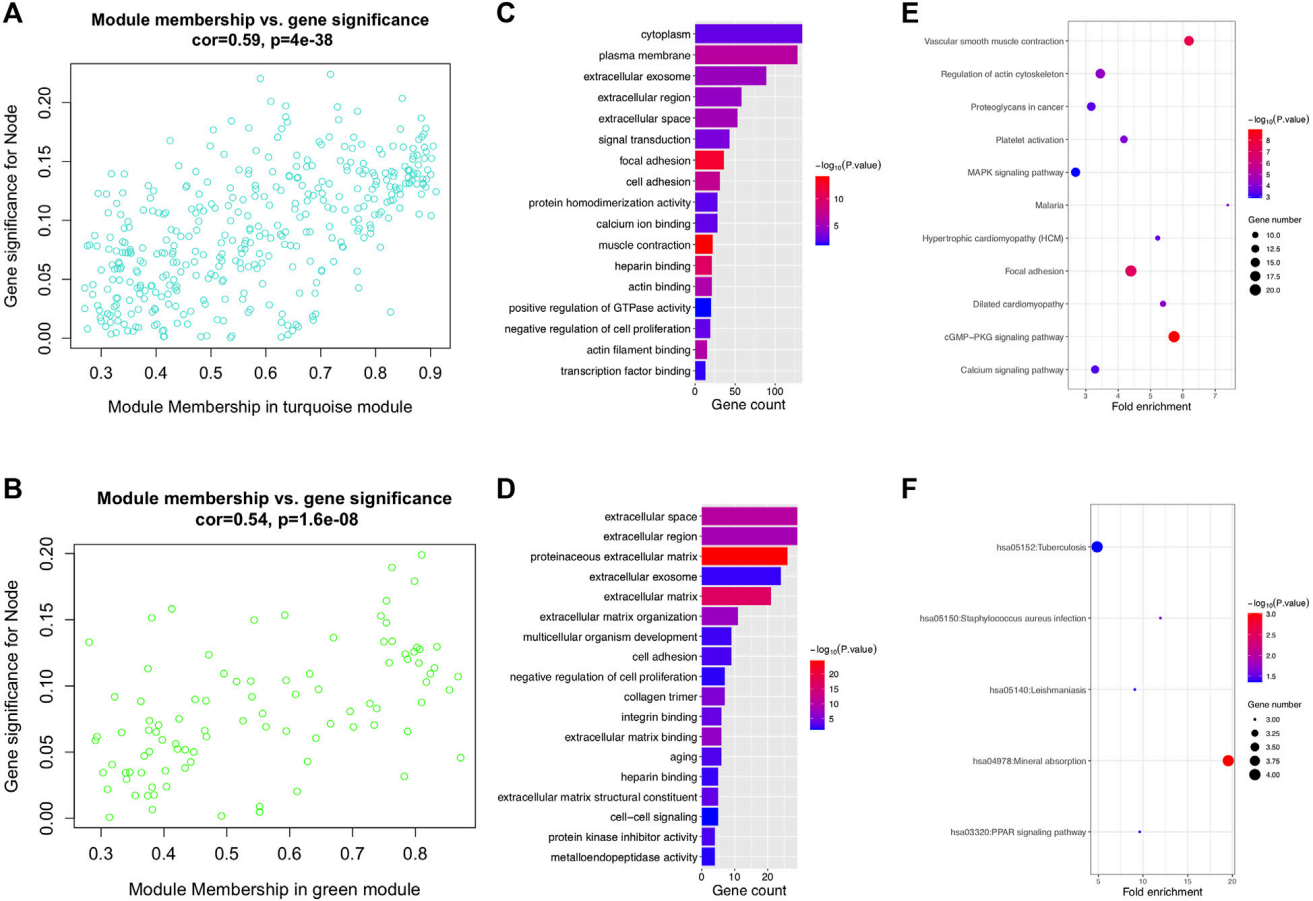
Lymph node metastasis–related modules and function analysis. **(A, B)** Scatterplot of module eigengenes in turquoise and green modules. **(C, D)** GO enrichment analysis of turquoise and green modules. **(E, F)** KEGG enrichment analysis of turquoise and green modules. GO: gene ontology and KEGG: Kyoto Encyclopedia of Genes and Genomes.

After differential analysis, 2,660 DEGs associated with LNM were identified and used for the construction of weighted co-expression network. The appropriate soft threshold power *β* was determined as three for subsequent adjacencies calculation (Figure 1A). Finally, nine gene modules were obtained on the basis of the results of the dynamic tree cut algorithm (Figure 1B). The correlation of module eigengenes was clustered and presented in Figure 1C, and to explore the clinical significance of co-expressed genes, the relationship of modules and clinical features were evaluated and visualized in Figure 1D, which showed that the turquoise and green modules were significantly associated with LNM of BCa. Furthermore, the correlation of module membership and gene significance in turquoise and green module are depicted in Figures 2A,B, respectively. We conducted the GO function and the KEGG pathway analyses to examine the potential function of the genes in turquoise and green modules. The GO analysis showed that the turquoise module was mainly enriched with cytoplasm, plasma membrane, and extracellular exosome (Figure 2C). Figure 2E showed the significant pathways including vascular muscle contraction, regulation of actin cytoskeleton, and proteoglycans in cancer. Similarly, Figures 2D,F presented the results of the GO and KEGG analyses of genes in the green module.

### Identification of Hub Genes and Results of GSEA

**FIGURE 3:**
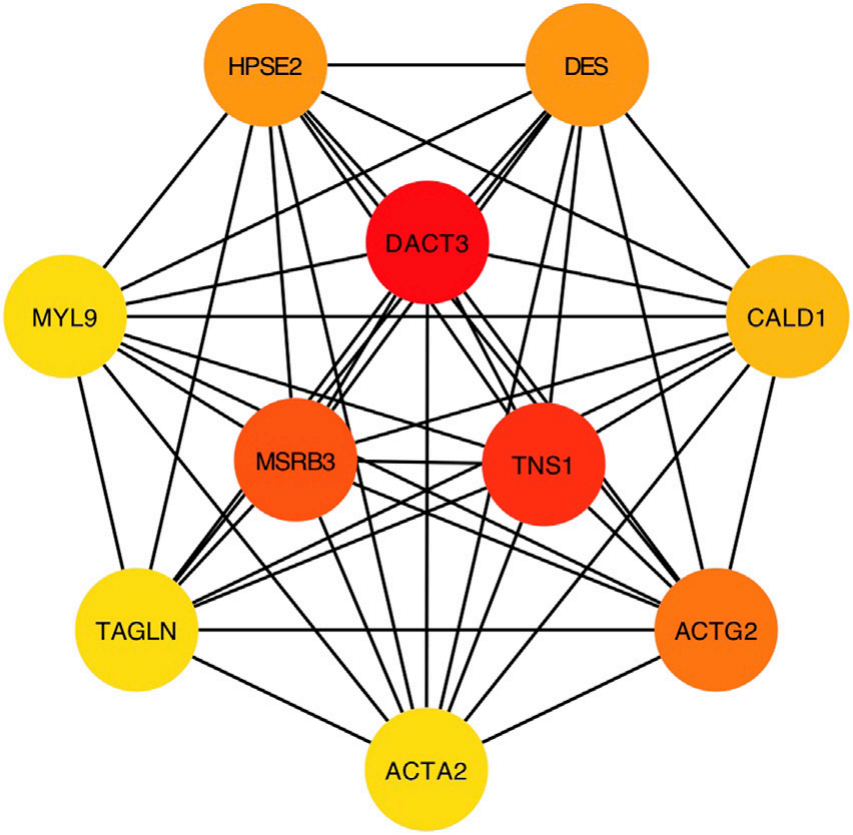
Network visualization of interested genes.

**FIGURE 4:**
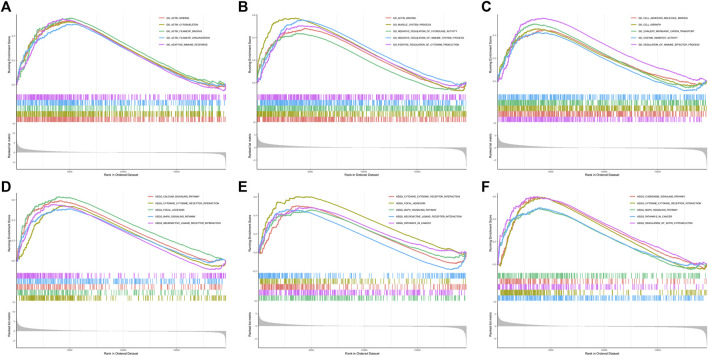
GSEA for DACT3, TNS1, and MSRB3. **(A–C)** GO enrichment analysis for DACT3, TNS1, and MSRB3. **(D–F)** KEGG enrichment analysis for DACT3, TNS1, and MSRB3. GSEA: gene set enrichment analysis.

Considering the most significant association of turquoise module and LNM, genes in the turquoise module were evaluated for the exploration of hub genes. The Cytoscape software was utilized for the construction of gene network. Figure 3 presented the network and the interaction of ten hub genes. Ranking by the degree method, top three hub genes were identified, including DACT3, TNS1, and MSRB3. Subsequently, GSEA was implemented for the GO and KEGG enrichment analyses to evaluate the biological function of the three hub genes. Figures 4A–C showed the GO analysis results, such as actin binding, actin cytoskeleton, adaptive immune response, and cell adhesion molecular binding. The KEGG pathways are available in Figures 4D–F, including calcium signaling pathway, cytokine receptor interaction, and focal adhesion. The corresponding normalized enrichment scores (NES) and the false discovery rate (FDR) *p*-values are listed in Supplementary Table 1.

### Clinical Significance of Hub Genes

**FIGURE 5:**
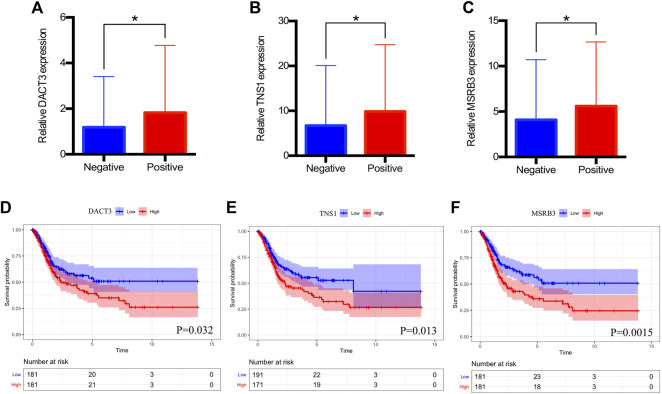
Clinical significance of DACT3, TNS1, and MSRB3. **(A–C)** Relative expression of DACT3, TNS1, and MSRB3 in patients with negative and positive lymph nodes. **(D–F)** Overall survival of patients with low and high expressions of DACT3, TNS1, and MSRB3. The asterisks mean *p* < 0.05.

The expression level of hub genes in negative and positive lymph node groups were compared. Figures 5A–C indicated the relative expression levels of DACT3 (*p* = 0.020), TNS1 (*p* = 0.040), and MSRB3 (*p* = 0.042) were significantly lower in the negative lymph node group than the levels of those in the positive group. Supplementary Figure 1 suggested that HPSE2, MYL9, TAGLN, CALD1, and ACTA2 were significantly associated with the lymph node status, except for DES and ACTG2. For prognostic impact of hub genes, high expression of DACT3 (*p* = 0.032, Figure 5D), TNS1 (*p* = 0.013, Figure 5E), and MSRB3 (*p* = 0.0015, Figure 5F) was significantly associated with a poor overall survival of BCa patients. In addition, the prognoses in groups with low and high expression levels of TAGLN, ACTA2, and CALD1 were significantly different (Supplementary Figure 2).

### Construction and Evaluation of Prognostic Signature

**FIGURE 6:**
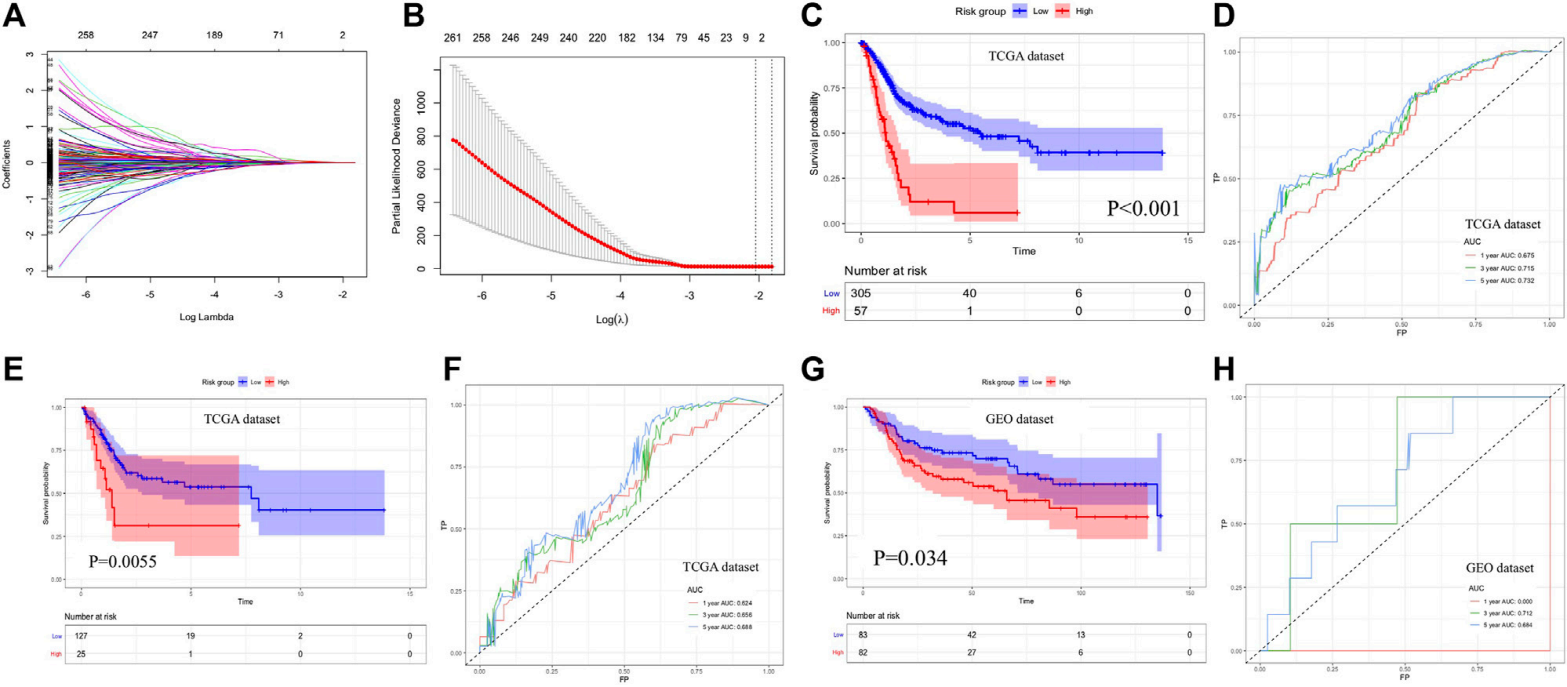
Construction and evaluation of prognostic signature. **(A, B)** LASSO regression analysis for screening candidate genes. **(C, D)** Overall survival analysis and time-dependent ROC curves of prognostic signature for predicting 1-year, 3-year, and 5-year survival in the TCGA database. **(E, F)** Survival analysis result and time-dependent ROC curves for internal validation. **(G, H)** Survival analysis result and time-dependent ROC curves for external validation with the GEO database. LASSO: least absolute shrinkage and selection operator, ROC: receiver operating characteristic, and GEO: gene expression omnibus.

 2By using the LASSO method, four genes including APOL2, AHNAK, GSDMB, and SHTN1 were screened out to construct the prognostic model (Figures 6A,B). Then, the multivariate COX regression analysis was performed to determine the coefficients of genes and construct the prognostic signature according to the previously described calculation. Based on the median value of the prognostic signature, patients were divided into two groups, and the survival rate was significantly lower in the high-risk group than in the low-risk group (Figure 6C). Time-dependent ROC indicated that the AUC values of prognostic signature for predicting 1-year, 3-year, and 5-year survival rate were 0.675, 0.715, and 0.732, respectively (Figure 6D). The prognostic signature was validated internally (*p* = 0.0055, Figure 6E) and externally (*p* = 0.034, Figure 6G) with good discrimination for survival. The ROC curves are presented in Figure 6F,H for validation with TCGA dataset and GEO dataset, respectively. The multivariate COX regression analysis indicated that the prognostic signature could independently predict the overall survival (HR: 3.185, 95% CI: 2.153–4.714, Table 1).

**TABLE 1:** Multivariate COX analysis of clinical parameters and prognostic signature.

	HR (95% CI)	p value
Age	1.755 (1.201–2.564)	0.004
Gender	1.258 (0.865–1.830)	0.230
Grade	0.001 (0.000–4.397e214)	0.965
Stage	2.350 (1.440–3.836)	0.001
Signature	3.185 (2.153–4.714)	<0.001

## Discussion

BCa is a complex malignancy with tremendous heterogeneity according to biological and histological features. To make an individualized therapeutic strategy, classification of BCa patient based on molecular subtypes by performing whole transcriptome profiling was reported previously. Luminal and basal subtypes, which were identified according to gene expressions, were associated with different response rates to chemotherapy as well as distinctly different prognosis of BCa patients (Dadhania et al., 2016; Choi et al., 2014). LNM is considered as one of the adverse pathological outcomes in association with poor prognosis (Sharma et al., 2019). Unfortunately, common pathological examination cannot detect the micrometastasis of lymph nodes. Gazquez et al. (2012) suggested that molecular biomarkers predicting LNM had a better detection rate than conventional histological evaluation.

Liu et al. (2021) summarized the coding genes regulating the LNM in bladder cancer, such as CCR7, PTBP-1, and UPK-1B with upregulated expression and GATA-6, NONO, and TCF-21 with downregulated expression in BCa tumor. However, the most relevant genes remained unclear, and the current study aimed to explore the biomarkers associated with LNM and prognostic indicators using bioinformatics analysis. WGCNA, an algorithm that constructs weighted co-expression networks, was applied to screen out meaningful modules related to interested clinical traits. In the present study, WGCNA was performed in the BCa dataset derived from the TCGA database. As a result, nine modules were constructed, and of them, green as well as turquoise modules were associated with LNM. Further enrichment analysis indicated that genes in the turquoise module were involved in the cGMP-PKG signaling pathway, vascular smooth muscle contraction, and focal adhesion. Genes in the green module were enriched in mineral absorption and the PPAR signaling pathway. These results implied that multiple biological processes participated in the progression and metastasis of BCa.

The hub genes were screened from the turquoise module, and the top three genes ranking by the degree were DACT3, TNS1, and MSRB3. DACT3 is a third DACT family member and has been revealed as a negative regulator of Wnt/*β*-catenin signaling, which plays an important role in cancer development (Fisher et al., 2006). It was reported that epigenetic reduction of DACT3 caused aberrant Wnt/*β*-catenin signaling in colorectal cancer cells and might be a potential pharmacological target for histone modification (Jiang et al., 2008). DACT3 was also demonstrated to inhibit the malignancy in other cancers such as non–small-cell lung cancer and esophageal squamous cell carcinoma (Zhao et al., 2017; Guo et al., 2017). However, the relationship between the function of DACT3 and the oncogenesis of BCa has not been studied. The present study suggested that DACT3 was strongly associated with the LNM of BCa, which needed further assessment and validation in the future studies. TNS1 was a member of tension family and demonstrated to bind to actin cytoskeleton and β1-integrin for adhesion-related signaling (Torr et al., 2015). It has been reported that TNS1 exerted an oncogenic role in the progression of several types of cancers *via* regulating the expression of genes related to cell motility (Zhou et al., 2016). Previous bioinformatics analysis indicated that TNS1 was the target of miRNA-31 and associated with the LNM of colorectal cancer (Zhang et al., 2019). MSRB3 has been recognized as one of the methionine sulfoxide reductase enzymes and plays an important role in cancer cell apoptosis. The reported mechanisms included protecting cancer cells from oxidative damage by eliminating reactive oxygen species, modulation of endoplasmic reticulum stress status, and participating in the malignant transformation of normal stem cells (Lim et al., 2012). In addition, MSRB3 was also demonstrated as a reliable predictor for metastasis in gastric cancer and prognosis in muscle-invasive BCa (Feng et al., 2020; Zhang et al., 2020).

To further investigate the functions and mechanisms of screened hub genes, GO and KEGG analyses using the GSEA algorithm were performed. As a result, the GO functions for DACT3, TNS1, and MSRB3 included actin binding, actin cytoskeleton, negative regulation of immune system process, and cell adhesion molecule binding, which were well-recognized biological processes for metastasis of cancers. The KEGG results revealed that the MAPK signaling pathway was annotated for these three hub genes. MAPK cascade is one of important signal pathways for the proliferation of cancer cells and a well-known signal pathway in the metastasis of BCa cells and might be a therapeutic target (Chao et al., 2019).

Furthermore, prognostic signature was constructed based on predictive genes, which were filtered from LNM-related modules including turquoise, green, and yellow modules by using the LASSO method. A previous study reported an outcome model for BCa based on WGCNA, and the respective accuracy of this model for predicting 1-year, 3-year, and 5-year survival rate was 0.626, 0.658, and 0.724 (Xiong et al., 2020), which was similar to our results. In the present study, four genes (APOL2, AHNAK, GSDMB, and SHTN1) were identified as significant factors to construct a prognostic model. The association between APOL2 and cancers was not widely explored. Recently, a 3’ untranslated region profile including APOL2 was reported to predict the LNM status in operative triple-negative breast cancer (Wang et al., 2019). AHNAK has been demonstrated as one of the cancer driver genes to participate in the tumorigenesis and progression of various malignancies, such as gastric cancer, prostate adenocarcinoma, and laryngeal cancer (Shen et al., 2020; Zhao et al., 2019; Dumitru et al., 2013). Moreover, recent studies suggested that AHNAK could be incorporated into immune-related signature for evaluating the prognosis of BCa patients (Qiu et al., 2020). The present study revealed that AHNAK was associated with the LNM and prognosis of BCa. GSDMB is a member of the gasdermin gene family and expressed in various types of cancer (Saeki et al., 2015). It was determined that the expression of GSDMB, which could be upregulated by interferon and mediated by pyroptosis in target cells (Zhou et al., 2020), was related to the response to HER2-targeted therapy and prognosis in breast cancer (Molina-Crespo et al., 2019). The present study constructed a prognostic signature based on LNM-related genes, and the signature showed a favorable prognostic value in training and validation datasets. However, the mechanisms of these genes in BCa were not thoroughly understood. Future research works were needed to evaluate the functions of hub genes and prognostic genes in BCa.

## Conclusion

In conclusion, the present study demonstrated that three hub genes (DACT3, TNS1, and MSRB3) were associated with the LNM of BCa by the WGCNA algorithm. Further, the KEGG analysis indicated that the MAPK signaling pathway was a common biological pathway for these three hub genes. In addition, based on genes from LNM-related modules, a prognostic signature was constructed and capable to predict the prognosis of BCa patients.

## Data Availability

The datasets presented in this study can be found in online repositories. The names of the repository/repositories and accession number(s) can be found in the article/Supplementary Material.

## Supplementary Material

Supplementary Figure 1 Relative expression of DES (A), HPSE2 (B), MYL9 (C), TAGLN (D), ACTG2 (E), CALD1 (F) and ACTA2 (G) in patients with negative and positive lymph nodes.

Supplementary Figure 2 Overall survival analysis results for DES (A), HPSE2 (B), MYL9 (C), TAGLN (D), ACTA2 (E), ACTG2 (F) and CALD1 (G).
