# Supplementary material for: Identification of Lymph Node Metastasis–Related Key Genes and Prognostic Risk Model in Bladder Cancer by Co-Expression Analysis
Source: Front Mol Biosci. 2021 Jul 22;8:633299. doi: 10.3389/fmolb.2021.633299 (PMC8339436; doi:10.3389/fmolb.2021.633299)
Supplement: Supplementary file 1 [file Table1.docx]

| Genes | Description | NES | FDR pvalue |
| --- | --- | --- | --- |
| DACT3 | GO_ACTIN_BINDING | 1.392112591 | 0.00990099 |
|  | GO_ACTIN_CYTOSKELETON | 1.377700506 | 0.00990099 |
|  | GO_ACTIN_FILAMENT_BINDING | 1.420026574 | 0.00990099 |
|  | GO_ACTIN_FILAMENT_ORGANIZATION | 1.32045715 | 0.00990099 |
|  | GO_ADAPTIVE_IMMUNE_RESPONSE | 1.429940213 | 0.00990099 |
| TNS1 | GO_ACTIN_BINDING | 1.497116461 | 0.00990099 |
|  | GO_MUSCLE_SYSTEM_PROCESS | 1.777893265 | 0.00990099 |
|  | GO_NEGATIVE_REGULATION_OF_HYDROLASE_ACTIVITY | 1.356723529 | 0.00990099 |
|  | GO_NEGATIVE_REGULATION_OF_IMMUNE_SYSTEM_PROCESS | 1.728480742 | 0.00990099 |
|  | GO_POSITIVE_REGULATION_OF_CYTOKINE_PRODUCTION | 1.571210086 | 0.00990099 |
| MSRB3 | GO_REGULATION_OF_IMMUNE_EFFECTOR_PROCESS | 1.844491227 | 0.01010101 |
|  | GO_CELL_ADHESION_MOLECULE_BINDING | 1.512172474 | 0.010204082 |
|  | GO_CELL_GROWTH | 1.531719947 | 0.010204082 |
|  | GO_DIVALENT_INORGANIC_CATION_TRANSPORT | 1.674654097 | 0.010204082 |
|  | GO_ENZYME_INHIBITOR_ACTIVITY | 1.416199954 | 0.010204082 |
| DACT3 | KEGG_CYTOKINE_CYTOKINE_RECEPTOR_INTERACTION | 1.454283057 | 0.00990099 |
|  | KEGG_FOCAL_ADHESION | 1.667246852 | 0.00990099 |
|  | KEGG_CALCIUM_SIGNALING_PATHWAY | 1.560029864 | 0.01 |
|  | KEGG_MAPK_SIGNALING_PATHWAY | 1.361546528 | 0.01 |
|  | KEGG_NEUROACTIVE_LIGAND_RECEPTOR_INTERACTION | 1.474526794 | 0.01 |
| TNS1 | KEGG_MAPK_SIGNALING_PATHWAY | 1.388755976 | 0.010752688 |
|  | KEGG_PATHWAYS_IN_CANCER | 1.476324332 | 0.010752688 |
|  | KEGG_CYTOKINE_CYTOKINE_RECEPTOR_INTERACTION | 1.521011999 | 0.010869565 |
|  | KEGG_FOCAL_ADHESION | 1.784133773 | 0.010869565 |
|  | KEGG_NEUROACTIVE_LIGAND_RECEPTOR_INTERACTION | 1.399097035 | 0.010869565 |
| MSRB3 | KEGG_PATHWAYS_IN_CANCER | 1.630772201 | 0.011235955 |
|  | KEGG_CYTOKINE_CYTOKINE_RECEPTOR_INTERACTION | 1.918128991 | 0.011494253 |
|  | KEGG_REGULATION_OF_ACTIN_CYTOSKELETON | 1.900143817 | 0.011494253 |
|  | KEGG_CHEMOKINE_SIGNALING_PATHWAY | 1.877192738 | 0.011627907 |
|  | KEGG_MAPK_SIGNALING_PATHWAY | 1.592356306 | 0.011627907 |

Supplementary table 1: The NES and FDR p value of the GSEA for hub genes

NES: normalized enrichment score; FDR: false discovery rate; GSEA: gene set enrichment analysis; GO: gene ontology, KEGG: Kyoto Encyclopedia of Genes and Genomes.
