# Supplementary figures and images for: Identification of Lymph Node Metastasis–Related Key Genes and Prognostic Risk Model in Bladder Cancer by Co-Expression Analysis
Source: Front Mol Biosci. 2021 Jul 22;8:633299. doi: 10.3389/fmolb.2021.633299 (PMC8339436; doi:10.3389/fmolb.2021.633299)

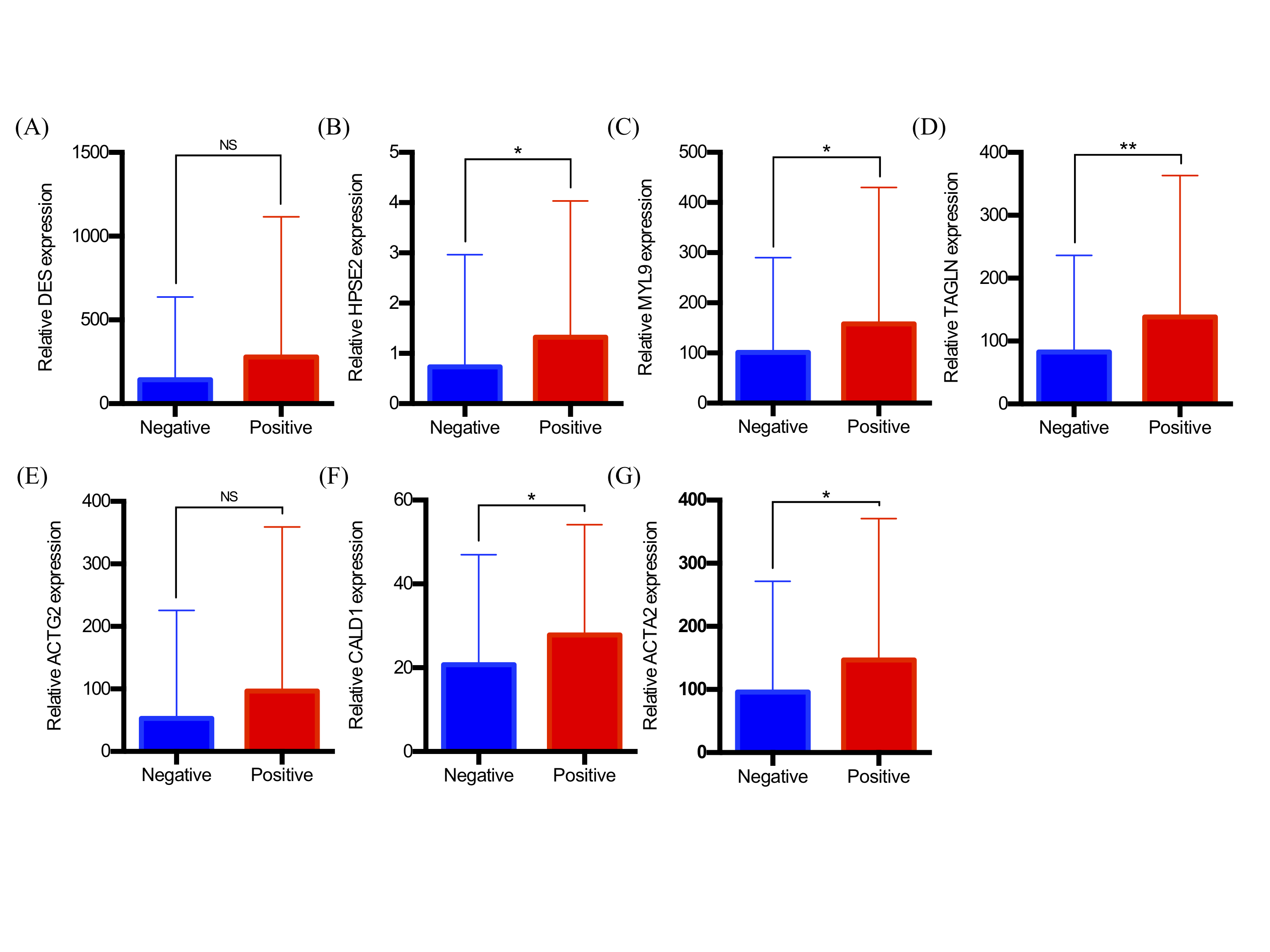

Supplement: Supplementary file 2 [file Image1.JPEG]

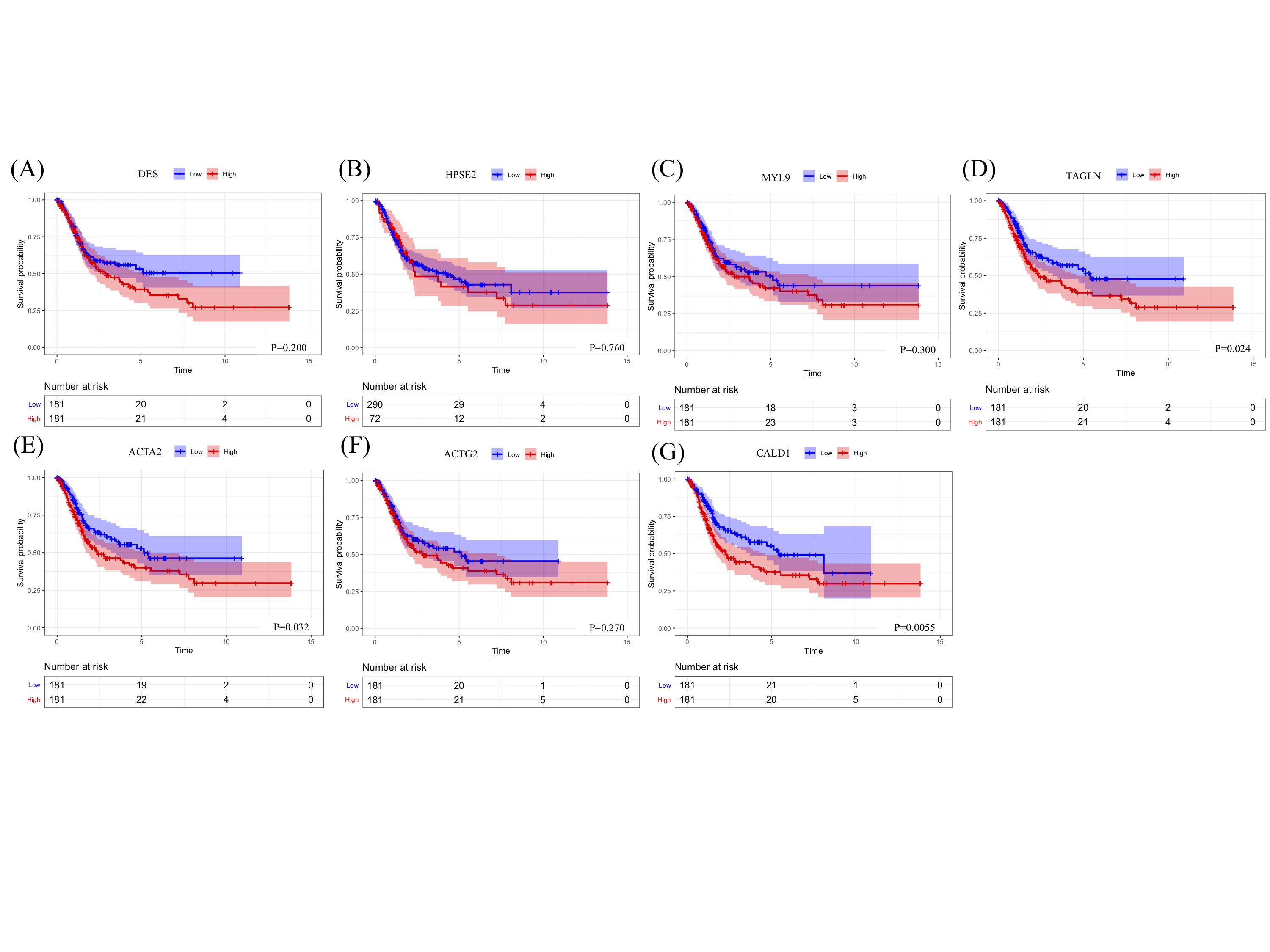

Supplement: Supplementary file 3 [file Image2.JPEG]
